# An Abnormally High Closing Potential of the OMPF Porin Channel from Yersinia Ruckeri: The Role of Charged Residues and Intramolecular Bonds

**DOI:** 10.32607/20758251-2019-11-3-89-98

**Published:** 2019

**Authors:** D. K. Chistyulin, O. D. Novikova, E. A. Zelepuga, V. A. Khomenko, G. N. Likhatskaya, O. Yu. Portnyagina, Y. N. Antonenko

**Affiliations:** Elyakov Pacific Institute of Bioorganic Chemistry, Far Eastern Branch, Russian Academy of Sciences, Prospect 100 let Vladivostoku 159, Vladivostok, 690022, Russia; Belozersky Institute of Physico-Chemical Biology, Lomonosov Moscow State University, Leninskie Gory 1/40, Moscow, 119991, Russia

**Keywords:** Yersinia ruckeri, pore-forming proteins, bilayer lipid membranes, voltage-dependent gating

## Abstract

Electrophysiological experiments on bilayer lipid membranes showed that the isolated outer membrane major porin of Yersinia ruckeri (YrOmpF) exhibits activity typical of porins from Gram-negative bacteria, forming channels with a mean conductance of 230 pS (in 0.1 M KCl) and slight asymmetry with respect to the applied voltage. Under acidic conditions (up to pH = 3.0), there was no significant decrease in the total conductance of the YrOmpF channel reconstituted into the bilayer. The studied channel significantly differed from the porins of other bacteria by high values of its critical closing potential (Vc): Vc = 232 mV at pH = 7.0 and Vc = 164 mV at pH = 5.0. A theoretical model of the YrOmpF spatial structure was used for the analysis of the charge distribution in the mouth and inside the channel to explain these properties and quantitatively assess the bonds between the amino acid residues in the L3 loop and on the inner wall of the barrel. The parameters of YrOmpF were compared with those of the classical OmpF porin from E. coli. The results of electrophysiological experiments and theoretical analysis are discussed in terms of the mechanism for voltage-dependent closing of porin channels.

## INTRODUCTION

Yersinia ruckeri is a Gram-negative bacterium that causes yersiniosis in fish, mainly in salmonids. Like other yersinia, this pathogen is able to survive and maintain virulence in various environmental conditions and in a wide temperature range. Y. ruckeri causes outbreaks of the disease in aquaculture fish, which leads to large economic losses each year [1-4].

Porins, along with lipopolysaccharide, are known to be a quantitatively dominant component of the outer membrane (OM) of Gram-negative bacteria and to play a crucial role in the adaptation of microorganisms to changing environmental conditions. Like transmembrane proteins, they form a system of channels for the passive transport of low-molecular-weight hydrophilic compounds through the bacterial OM. The main functional unit of porins is a homotrimer [5, 6]. The protein monomer is an ellipsoid beta-folded cylinder (barrel) consisting of antiparallel beta strands connected by segments (external loops) with an alpha-helical or disordered structure. The inner part of the porin monomer channel is the hydrophilic surface of the beta-barrel, and the outer part is formed by adjacent parts of the loops (the pore mouth and vestibule region). The pore vestibule is in immediate contact with a fragment of the adjacent barrel’s loop L2 that is directed away from “its” monomer. In the channel center, there is the longest loop L3 that, unlike the others extending outside the barrel, is inserted into the middle of the pore, thereby limiting its size and forming a narrowing, the so-called constriction zone or pore eyelet. The barrel wall consists mainly of positively charged amino acid (AA) residues; on the contrary, the L3 loop contains a large number of acidic AA residues. The spatial configuration of charged AA residues is such that an electrostatic field is generated inside the channel and underlies the channel’s selectivity for the charges of penetrating ions and hydrophilic compounds [7].

Electrophysiological experiments performed on nonspecific porins from Escherichia coli have demonstrated that the OmpF protein channel occurs in an open state most of the time, ensuring the entry of ions and hydrophilic molecules into the cell. However, most porins can switch to a stable, closed state; e.g., upon increasing medium acidity and/or under an applied external voltage (voltage-dependent closure) [8-14].

With regard to the biological function of these channel properties, various hypotheses are put forward. In particular, this may be due to the closing of the channels of improperly incorporated proteins and may also be the protective (as the medium pH decreases) or even regulatory transport function of porins (e.g., in proteins with a very low critical channel closure voltage, Vc) [15, 16]. However, all the proposed explanations are not sufficiently convincing and, perhaps, this property may be considered only as an unusual artifact [17].

Various suggestions for the mechanism of voltagedependent closure of porin channels (gating mechanism) have been proposed. Based on molecular dynamics (MD) data, a model of a movable loop L3 whose displacement leads to channel blockage was proposed as a possible gating mechanism [18]. However, because this loop has many interactions with the barrel wall (salt bridges, hydrogen bond network), this idea seems unlikely. In addition, the closing of the channel is not accompanied by significant changes in the loop’s position: there are no noticeable differences in this property in E. coli OmpF whose L3 loop is modified with disulfide bridges [19, 20]. This fact indicates that a potential cause of channel blockage may be local changes in the tertiary structure of some L3 loop fragments. MD studies of perturbations have suggested that at least part of loop L3 from R. capsulatus porin is flexible [21]. This part may well correspond to the region immediately following the conserved PEFGG sequence motif found in OmpF from Escherichia coli. Indeed, replacement of two glycine residues in PEFGG led to a change in the functional properties of the channel [22]. It is worth noting that the hypotheses explaining the voltage-dependent closing of the channels of the poreforming proteins, as well as the facts underlying those hypotheses, are quite contradictory. For example, the charged AA residues located inside the barrel and generating the electrostatic field are known to strongly affect the Vc value. Moreover, the replacement of negatively and positively charged residues with neutral ones has a different effect on porins of different types. For example, PhoE from E. coli, which is selective for negative ions, decreases Vc in response to a substitution of acidic residues in the L3 loop by neutral ones, while cation-selective OmpF from E. coli increases Vc. On the contrary, substitution of basic residues in the barrel increases Vc in PhoE and decreases Vc in OmpF [23].

However, based on the hypothesis of a flexible loop L3, the inconsistency of experimental facts may be explained by a dual role of charged AA residues. On the one hand, these residues are involved, through hydrogen and ionic bonds, with neighboring AA residues in the channel tertiary structure formation and, therefore, in the stabilization of the channel open state. On the other hand, they are sensors of the electric field and promote the transition of the channel to a closed state. In this case, their sensitivity to changes in the membrane potential, in combination with localization in the long and rather mobile L3 loop, may cause conformational changes in the L3 loop. This is explained by the fact that the transport of molecules through the pore is accompanied by a redistribution of water molecules (or counterions) inside the channel and a related reorientation of the side chains of AA residues in the channel. As a result, there may be local displacements within the L3 loop, which could lead to closure of the pore [13, 24].

In this work, we characterized the electrophysiological properties of the porin channels OmpF from the OM of Y. ruckeri (YrOmpF) and OmpF from E. coli (EcOmpF) using artificial bilayer lipid membranes (BLMs); namely, we determined single channel conductance for these proteins and critical closing potentials in neutral and slightly acidic media. We also investigated the changes in the total conductance of the channels during stepwise changes in the medium pH to a pH of 3.0. We used spatial models of YrOmpF and EcOmpF for a comparative analysis of the charged AA distribution in the mouth, vestibule, and inside the channel of both proteins, as well as for a quantitative assessment of the intramolecular bonds within the L3 loop. Given the decisive importance of these data for characterizing the functional properties of porin channels, this comparison was of particular interest, because the OmpF porin from Y. ruckeri differs in its number of acidic AA residues in the L3 loop from the classical OmpF porin of E. coli. The calculated data enabled the identification of a more rigid L3 loop conformation in YrOmpF, which obviously affects the open state stability of its channel and underlies the higher Vc value.

## EXPERIMENTAL

**Microorganisms**

Y. ruckeri (strain KMM 821) was used in the study. Microorganisms were cultured in 2×YT medium at 6 °C as described in [25] and were harvested at the logarithmic growth phase. Then, the cell suspension was centrifuged at 5,000 g and the resulting pellet was washed twice with physiological saline.

**Preparation of peptidoglycan-associated protein fractions and isolation and purification of YrOmpF porin**

Y. ruckeri bacteria were destroyed by ultrasound using a disintegrator (UZDN-2T, Russia) at 44 MHz (10 times for 1 min with a 1–2 min break to cool the mixture) in an ice bath. Undisrupted cells were removed by centrifugation at 5,000 g for 10 min, and the supernatant was centrifuged at 20,000 g for 1 h. The resulting crude membrane fraction in the form of a pellet was treated with 0.5% nonionic detergent n-octyl-polyoxyethylene (POE) in 10 mM phosphate buffer pH 8.5 (buffer A), according to the Garavito procedure [26]. The target protein in the extracts was determined by denaturing polyacrylamide gel electrophoresis (SDS-PAGE) [27]. Fractions containing maximum amounts of oligomeric YrOmpF were pooled and purified by ion exchange chromatography on DEAE-Sepharose CL 6B; the protein was eluted with buffer A containing 0.1% Zwittergent 3-14, using a 0.137–0.5 M NaCl gradient. Homogeneous electrophoretically pure YrOmpF was eluted with 0.4 M NaCl, which was confirmed by SDS-PAGE. This sample was used in the electrophysiological experiments.

**Electrophysiological experiments**

BLMs were prepared according to the Mueller–Rudin technique [28] from a diphytanoylphosphatidyl choline (DPhPC) solution in n-heptane (5 mg/mL) in Teflon cells separated by a septum with 1-mm holes for the total current and 0.25-mm holes for single channels. The aqueous phase contained 0.1 or 1 M KCl in the following buffer: 10 mM Tris-HCl, 10 mM MES, and 10 mM beta-alanine (pH 7.0, 5.0, and 2.8). The ion current was detected by a pair of Ag/AgCl electrodes in voltage detection mode. The electrode on the cis membrane side was grounded, and that on the trans side was connected to a BC-525C amplifier (Warner Instrunments, USA). Measurements were carried out at room temperature. A protein solution was added to the cis side of the cell, and, by raising the voltage to 200 mV, channels were inserted. The total current through the BLM was recorded at a YrOmpF concentration of 50–500 ng/mL; single protein channels were obtained at a concentration of 5–20 ng/mL. Changes in the current through the BLM were recorded in the presence of the protein dissolved in neutral or acidic buffer at various membrane potential values (50 to 150 mV).

**Theoretical analysis of intramolecular bonds**

To generate a theoretical model of the spatial Y. ruckeri OmpF structure, we used the AA sequence of porin E2FHC9 from the Uniprot database [29]; the atomic coordinates of E. coli OmpF porin (PDB ID 2OMF) were used as a prototype. Homologous models were generated using the MOE software as described previously [30]. The models were optimized with the MOE 2018.0101 program and Amber10:EHT force field [31]. According to the Ramachandran map, about 96.4% of residues in the generated models of the YrOmpF and EcOmpF channels occurred in a favorable conformation and 3.6% of residues were in an allowable conformation. This indicated that these models might be used for further investigation. The energy contribution of intramolecular non-covalent interactions to the porin structure formation was analyzed and evaluated using the MOE 2018.0101 program [31]. The geometric and physicochemical parameters of the pore’s interior were estimated using a distant MOLE online resource [32].

## RESULTS AND DISCUSSION

**Electrophysiological properties of YrOmpF in neutral and acidic media**

**Fig. 1:**
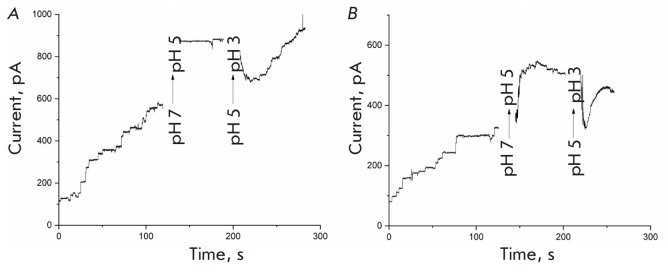
Channel conductance of Y. ruckeri OmpF (YrOmpF) and E. coli OmpF (EcOmpF) porins when changing pH from 7.0 to 3.0. Aqueous phase: 0.1M KCl, 10 mM Tris-HCl, 10 mM MES, 10 mM beta-alanine, 100 ng/mL protein. Voltage, 50 mV. A – EcOmpF; B – YrOmpF

Figure 1 shows changes in the total electrical conductance of a planar DPhPC bilayer membrane under a voltage of ± 50 mV in the presence of YrOmpF or EcOmpF at various pH values. The current fluctuations (initial parts of the curves) illustrate an active stepwise increase in the membrane conductance upon addition of porins at a concentration of 100 ng/mL into the aqueous phase (buffer pH 7.0). This effect, characteristic of Gram-negative bacterial porins, reflects the incorporation of functionally active protein trimers.

To evaluate the potential effect of medium acidity on the functional activity of YrOmpF reconstituted in the lipid bilayer, the aqueous phase in both parts of the cell was sequentially acidified to pH 5.0 and 3.0 during the experiment. The current recordings shown in Fig. 1B (second and third segments) demonstrate that as the medium pH decreases, the membrane conductance increases. In this case, the single channel conductance does not change, which means that this effect illustrates a sharp activation of protein incorporation into the membrane.

At a medium pH of 3.0 (the third curve segment), the total conductance of the YrOmpF channel sharply decreases and then gradually recovers. Current recordings under these conditions are characterized by increased noise, which is typical of porin channels in an extremely acidic medium and is associated with fast opening/closing of channels.

It should be noted that we did not observe a decrease in the total channel conductance during short-term incubation (for minutes) of the protein at low pHs. However, long-term incubation of the protein in buffer at pH 3.0 before incorporation into the BLM led to a loss in the functional activity of YrOmpF, which was not restored even after neutralization of the medium (data not shown). Probably, at extremely acidic medium pHs, the studied porin molecules undergo significant conformational changes disabling porins to form conducting channels in the membrane. Obviously, the lipid environment protects the protein from similar changes in the spatial structure and facilitates the stabilization of their functionally active conformation, which leads to preservation of the functional activity of most of the incorporated channels.

To investigate the effect of pH on the conductance of a single YrOmpF channel and its asymmetry, the protein (10 ng/mL) was inserted into the membrane in buffer at pH 7.0 in 0.1 M KCl then the buffer was acidified in both cells simultaneously. During this experiment, the porin channel was found to have a small conductance asymmetry (12%), which remained during medium acidification to pH 5.0. The channel conductance during acidification increased by 2% (n = 4), on average. It should be noted that a similar channel asymmetry was also observed for the E. coli OmpF porin [14].

**Fig. 2:**
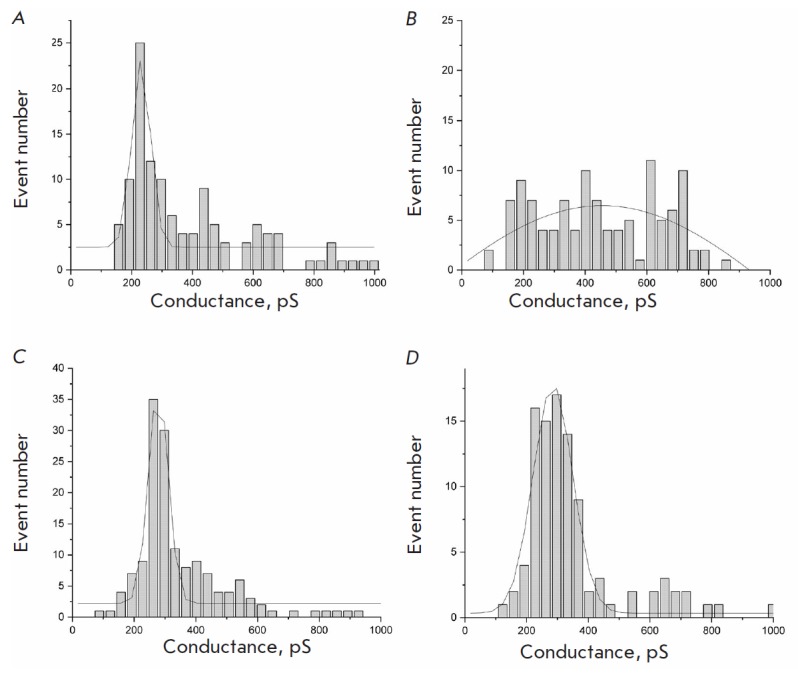
Distribution of single channel conductance of YrOmpF and EcOmpF in a DPh- PC BLM. The proteins were reconstituted at neutral pH = 7.0 (A) and acidic pH = 5.0 (B). Aqueous phase: 0.1M KCl, 10 mM Tris-HCl, 10 mM MES, 10 mM beta-alanine, 100 ng/mL protein. Voltage, 50–150 mV

The conductance histograms of single YrOmpF and EcOmpF channels in neutral and acidic media (Fig. 2) were obtained in 0.1 M KCl. Protein samples pre-incubated in buffer solutions with different pHs (7.0 and 5.0) were added to the cis side of the cell to a final concentration of 100 ng/mL, and a voltage of 50 to 150 mV was applied. During the experiment, hundreds of insertional steps of the studied proteins were analyzed.

Inserted into the model DPhPC membrane both in neutral and acidic media, YrOmpF was shown to form a pore population heterogeneous in conductance. At pH 7.0 in 0.1 M KCl, the largest number of channels had a conductance of about 230 pS (Fig. 2A); in this case, the histogram contains minor multiple conductance peaks, which are obviously associated with protein trimer aggregates (460 and 690 pS). As the medium pH decreased to 5.0 (Fig. 2B), the conductance heterogeneity of YrOmpF channels increased even more. Additional peaks appeared on the histogram, and the proportion of channels with major multiple conductance also increased.

Compared to YrOmpF, the EcOmpF channel is characterized by a less heterogeneous pore population with a peak of 276 pS at pH 7.0 and 285 pS at pH 5.0. However, in an acidic medium, a wider distribution of channel conductance and EcOmpF protein is observed.

Previously, we demonstrated that the Yersinia porins, especially nonpathogenic ones [33], were characterized by a wide range of channel conductance levels compared to E. coli OmpF. In the case of YrOmpF, this may be due to the fact that this porin is a wild-type protein obtained from the membrane using the nonionic POE detergent that more gently affects the porin conformation upon release than the ionic SDS detergent. For this reason, protein trimer associates with a higher conductance may remain in the YrOmpF sample. The described pH-dependent changes in the functional properties of YrOmpF were also observed in the OmpF channels from Y. pseudotuberculosis (YpOmpF). We found that the protein occurred predominantly as a trimer in the aqueous solution at pH 7.0 and as a monomer at pH 3.0 [34]. The main disturbances in the spatial organization of YpOmpF in an acidic medium are associated with a decrease in the beta-barrel packing density and the changes in the microenvironment of the aromatic chromophores in the protein molecule. At low pHs, changes in the electrostatic potential on the protein surface are accompanied by significant structural rearrangements, which leads to dissociation of porin trimers into monomers [34]. In addition, we showed earlier that both molecular protein forms (trimer and monomer) had high affinity for the membrane, but only binding of trimers led to porin channel formation in the lipid bilayer [35].

Therefore, the experimental data obtained for YrOmpF and EcOmpF and the results of earlier studies of a closely related porin of the pseudotuberculosis microbe suggest that extremely low pHs lead to irreversible changes in the ability of the studied porins to incorporate into the model membrane to form channels. However, these conditions do not reduce the conductance of pre-inserted channels. Therefore, the tendency of porin channels to close at lower pH is unlikely to play a significant role in the regulation of ion fluxes through the bacterial membrane.

**Potential-dependent closing of YrOmpF channels**

One of the properties of pore-forming protein channels from Gram-negative bacteria is their ability to switch to a closed state as the voltage applied to the membrane is increased. This closing is stepwise and reflects sequential closure of monomer channels in the protein trimer.

Because YrOmpF channels had a weak tendency to close, and high membrane potentials (more than 220 mV) often led to significant activation of channel incorporation, recording of classical current–voltage characteristics posed certain experimental difficulties. Therefore, the ability of YrOmpF channels for voltagedependent closure was studied in single channels. For this purpose, a 5 ng/mL YrOmpF sample was added to the cell cis side, and the membrane potential was increased to 250 mV, awaiting a single channel insertion event. Then, the voltage was reduced to 100 mV and increased stepwise at a rate of 10 mV/min. The voltage causing stable closure of at least one monomer was considered the critical closing voltage (Vc). Similarly, 10 YrOmpF channels were analyzed at pH 7.0 and 15 channels at pH 5.0, which enabled to measure the Vc value under these conditions. We also used this technique for measuring Vc of the EcOmpF channels. The obtained values are given in Table.

**Table 1:** Critical closing potential of the studied porins

Porin	Vc, mV	
	pH 7.0	pH 5.0
E. coli OmpF	124 ± 6 (n = 10)	103 ± 10 (n = 15)
Y. ruckeri OmpF	232 ± 7 (n = 10)	164 ± 8 (n = 15)

**Fig. 3:**
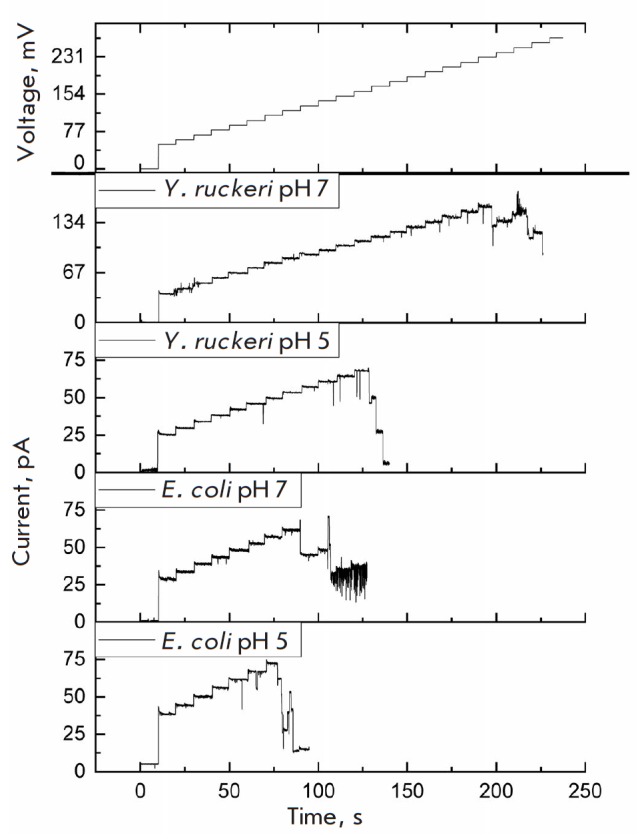
Conductance of YrOmpF and EcOmpF porin channels during a stepwise increase in the membrane potential. Aqueous phase: 0.1M KCl, 10 mM Tris-HCl, 10 mM MES, 10 mM beta-alanine, 10 ng/mL protein. Voltage, 0, 50, –250 mV

Typical current recordings illustrating the difference in the closing voltage of the channels of the two proteins are shown in Fig. 3 (not all channels in the given records are single).

During the experiment, YrOmpF channels were found to have unusually high critical closing potentials compared to those of EcOmpF channels (Table). In addition, this characteristic of the YrOmpF channels was found to depend on medium acidity because lowering of the electrolyte pH to 5.0 led to a decrease in Vc. Thus, the pH dependence of channel conductance for the studied protein is similar to that for the E. coli OmpF channels [36]. The Vc values obtained for the EcOmpF sample used in this study also correspond to the data of [36].

The functional characteristics of porin channels are known to be controlled mainly by the structure of their constriction region, where the beta-barrel diameter decreases significantly [7]. An unusual organization of the pore constriction region with two oppositely charged semirings situated across each other in a restricted space generates an intense electrostatic field in the pore, which controls solute flow through the channel and determines the pore activity of a given protein.

The cationic cluster on the inner wall of the E. coli OmpF barrel is formed by three arginine residues (Arg42, Arg82, and Arg132) that are flanked by a lysine residue (Lys16). A positively charged cluster is present inside the OmpF barrel of Yersinia porins, like in E. coli OmpF as was shown earlier [37]. In YrOmpF, this arginine cluster is represented by three residues (Arg37, Arg76, and Arg127). However, the acidic residue Glu117 (present in E. coli OmpF) in a highly conserved PEFGG porin region of the loop L3 [38] is replaced by neutral Val111 as in other Yersinia. In addition, this loop in YrOmpF lacks another charged residue: Asp127 (in E. coli OmpF) is replaced by Asn122. As a result, instead of six acidic residues in the L3 loop of E. coli OmpF, YrOmpF contains only four residues, whose charge can change in an acidic medium.

Replacement of charged residues in the L3 loop and in opposite segments of the beta-barrel in the AA sequence of E. coli OmpF is known to lead to significant Vc variations. For example, higher Vc values were obtained for E. coli OmpF mutants with acidic residues in the L3 loop replaced by neutral ones [23]. Therefore, the structural differences in the functionally important sites of the L3 loop, revealed by a comparative analysis of the AA sequences of YrOmpF and OmpF from E. coli, may be responsible for the differences in the Vc values of these two proteins.

**Analysis of intramolecular interactions based on theoretical porin models**

To explain the higher experimental Vc value of Y. ruckeri OmpF porin compared to that of classical E. coli OmpF, we used a comparative analysis of the charge distribution at the mouth, entrance, and inside the pore in a theoretical model of the spatial structure of these two proteins, which was generated by homologous modeling.

Alignment of the AA sequences of the studied proteins revealed that the primary structure of the barrel in their molecules has a high degree of homology, but that the external loops differ in the length and AA composition. Here, there are both inclusions of additional residues and deletions. For example, loop L1 in YrOmpF is shorter by two residues, and loops L4 and L8 contain two and four additional residues, respectively, compared to the same loops in EcOmpF. In addition to the differences in the number of AA residues in the loops forming the channel entrance, the number of basic residues in this region of the EcOmpF molecule was found to be noticeably smaller than that in YrOmpF (data not shown).

**Fig. 4:**
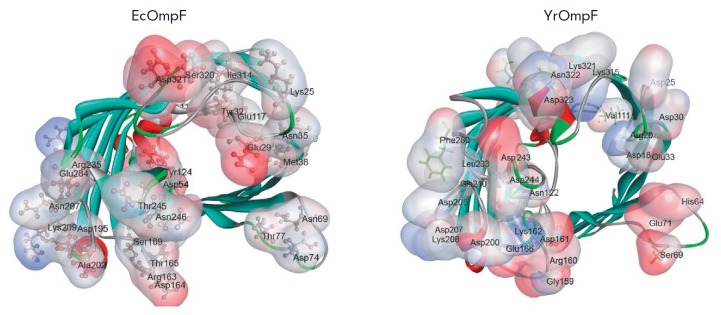
The ...

**Fig. 5:**
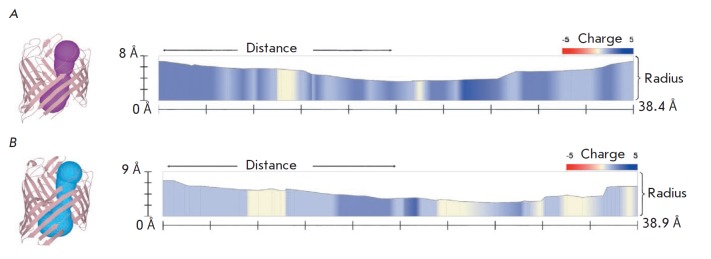
The ...

A detailed MOLE-based analysis of the charge distribution revealed significant differences in the number and localization of basic and acidic AA residues both in the external loop region and inside the pore of the studied proteins (Figure 4 and Figure 5). For example, the channel entrance region in EcOmpF contains a greater amount of acidic AA residues (Fig. 4), so this region is charged more negatively than that in YrOmpF. However, the external vestibule and constriction zone of the EcOmpF channel contain more basic AA residues and, therefore, have a stronger positive charge than YrOmpF (Fig. 5).

In addition, despite a comparable pore length (38.9 Å for YrOmpF and 38.4 Å for EcOmpF), the studied porins significantly differ in their charge distribution in the inner part of their channels. For example, the interior of EcOmpF is characterized by a finer structural organization in terms of alternating positively and negatively charged residues along the pore, while the inner surface of the YrOmpF channel contains longer charged areas (Fig. 5).

These facts may be one of the causes for the differences in the closing potential of the studied proteins. It is known that Omp-Pst1 and Omp-Pst2 porins from Providencia stuartii, which have close structural similarity but significantly differ in their charge distribution patterns along channel walls and, respectively, in the strength of electrostatic interactions inside the pore, not only possess opposite ion selectivity, but also significantly differ in their closing potential [16].

On the other hand, the degree of conformational mobility of the L3 loop is known to be controlled by the network of hydrogen bonds and salt bridges located between the top and base of L3 and the adjacent barrel wall [39]. It is the strength of these bonds that affects the porin channel sensitivity to the membrane potential [16]. Therefore, the features of intramolecular interactions associated with L3 may play a significant role not only in the pore conductance, but also in the voltage-gated switching of the channel on/off. As mentioned above, the hypothesis of a “flexible” L3 loop is the most plausible among existing explanations for voltage-gating of porin channels. Due to its capacity for significant fluctuations, this loop can change its spatial orientation under voltage applied to the membrane, which switches off the ion flow. If this hypothesis is true, then the difference in the closing potential between YrOmpF and EcOmpF porins should depend on the degree of conformation stability of the L3 loop. In both proteins, this is controlled by the contacts and bonds that exist between specific AA residues in the L3 loop and residues in other loops and the barrel wall.

An analysis of the intramolecular interactions between the residues of the L3 loop in the studied porins revealed that the total interaction number varies significantly. For example, the position of this loop in EcOmpF is stabilized by 23 non-covalent interactions with a total energy contribution of –63.8 kcal/ mol, while the L3 loop conformation in YrOmpF is controlled by 35 interactions whose energy is –131.6 kcal/mol.

**Fig. 6:**
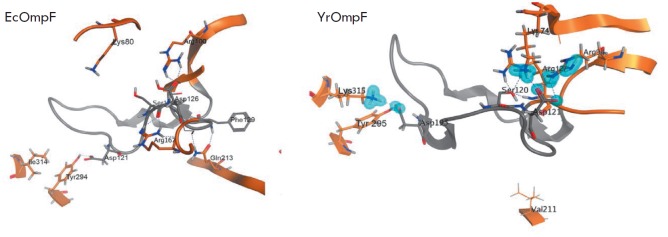
The ...

Intramolecular non-covalent interactions of functionally important amino acid residues in loop L3 in YrOmpF and EcOmpF porins. Protein secondary-structure elements are shown as ribbons; functionally important amino acid residues are shown as sticks. Elements of loop L3 are shown in grey; β-strands and other loops are shown in brown. Hydrogen bonds are shown as blue dotted lines; ionic interactions are shown as blue contours in EcOmpF, forms five salt bridges and four hydrogen bonds with Arg94 in the β5 strand (corresponds to Arg100 in EcOmpF) with a total contribution of about –17.563 kcal/mol. In addition, there is another hydrogen bond between Asp121 and Lys74 in the β4 strand of Y. ruckeri porin (Lys80 in EcOmpF) with a contribution of –13.8 kcal/mol (Fig. 6).

In another part of the L3 loop, replacement of Ile314 (in EcOmpF) with Lys315 (in YrOmpF) changes the interaction pattern of the conserved Asp115 residue (corresponds to Asp121 in EcOmpF) in the L3 loop of YrOmpF porin. Therefore, in addition to the interactions between Asp115 and Tyr295 (–38.27 kcal/mol) conservative for these porins, Asp115 in YrOmpF forms a network of energy-intensive hydrogen bonds and ionic interactions (–10, –6.355, and –2.652 kcal/mol) with the Lys315 side chain in the β15 strand, which are absent in EcOmpF (Fig. 6).

Therefore, the calculated data indicate that the L3 loop of YrOmpF has a more stable conformation.

## CONCLUSIONS

Our electrophysiological experiments revealed an abnormally high critical closing potential of the OmpF channel from Y. ruckeri compared to that of the E. coli porin. A theoretical analysis of the charge distribution in regions of the spatial porin structure which are important for channel conductance and a quantitative assessment of the intramolecular bonds inside the YrOmpF and EcOmpF pores revealed significant differences in polar interactions between the AA residues of the L3 loop and the barrel. The conformational mobility of the L3 loop in YrOmpF is much more restricted, which may create a need to apply an additional (compared to E. coli porin) potential in order to switch the YrOmpF channel to a closed state.

The obtained results contribute to the investigation of the molecular mechanisms of channel conductance in nonspecific porins from Gram-negative bacteria. These proteins are of interest, as biological nanopores, for use in nanotechnology and nanomedicine. The basis for this is their ability to change conductivity in response to any external factor and/or an analyte. In this regard, a detailed investigation of the structural basis for the functioning of pore-forming proteins will lead to a more meaningful approach to the design of biological sensors with the desired properties.

## Abbreviations

YrOmpF: Yersinia ruckeri OmpF porin
EcOmpF: Escherichia coli OmpF porin
Vc: critical voltage
OM: outer membrane
AA: amino acid
MD: molecular dynamics
BLM: bilayer lipid membrane
octyl-POE: n-octylpolyoxyethylene
DPhPC: 1,2-diphytanoyl-sn-glycero-3-phosphocholine
